# Why Size Does Not Matter: Sex Driven Home Range Differences in Brown Bears

**DOI:** 10.1002/ece3.73531

**Published:** 2026-04-20

**Authors:** Morteza Naderi, Emrah Çoban, Uygar Can Çelik, Josip Kusak, Çağan H. Şekercioğlu

**Affiliations:** ^1^ Department of Biology, Faculty of Sciences Sakarya University Sakarya Türkiye; ^2^ KuzeyDoğa Derneği Kars Türkiye; ^3^ Veterinary Biology Unit, Faculty of Veterinary Medicine University of Zagreb Zagreb Croatia; ^4^ Department of Molecular Biology and Genetics Koç University Istanbul Türkiye; ^5^ School of Biological Sciences University of Utah Salt Lake City Utah USA

**Keywords:** body size, brown bear, GPS telemetry, home range, movement ecology, principal component analysis, sexual dimorphism, space use

## Abstract

Sex, body size and environmental conditions are key determinants of home range size in large mammals, yet their relative importance within populations remains unclear for many wide‐ranging carnivores such as brown bears. This study combined GPS‐collar data and detailed morphometrics from 69 free‐ranging brown bears (46 males, 23 females) in Türkiye to test how home range size relates to sex, body mass, and body dimensions. Individual home ranges were estimated using 95% minimum convex polygon (MCP95) and kernel utilisation distribution methods, and the MCP95 area was log‐transformed and analysed with linear models that included sex, log body mass, representative linear size measures, sampling effort covariates, and principal component scores derived from a multivariate analysis of eight morphometric traits. Home range size varied markedly among individuals (5.1–203.9 km^2^), with males consistently having nearly twice the MCP95 area of females, with sex accounting for most of the variance in log home range size. In contrast, neither body mass nor linear body dimensions showed significant within‐sex relationships with home range size, and sex × mass interactions were not supported. Principal component analysis revealed a dominant body size axis, but this multivariate size component, as well as a secondary shape axis, did not explain additional variation once sex was included. Models that accounted for the number of GPS fixes, tracking duration, and influential points confirmed the robustness of the sex effect and indicated only modest contributions of sampling effort. Overall, the results showed that sex, rather than fine‐scale variation in body size or shape, is the primary intrinsic driver of home range size in this southern brown bear population, highlighting the need for sex‐specific but not size‐specific spatial planning and conservation strategies in the future.

## Graphical abstract

This is a novel anlaysis of unique dataset never find anywhere more than 60 collared free ranging brown bears movement data.

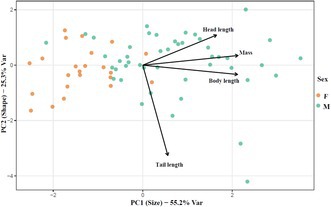

## 1 Introduction

Home range size in brown bears varies widely among individuals and populations and is shaped by sex, body size, reproductive status, resource availability, and landscape structure (Reiss 1988; Dahle and Swenson 2003; Ofstad et al. 2016; Joly et al. 2022). Males typically have substantially larger home ranges than females because they range widely to locate receptive females and defend access to them. Among females, reproductive status strongly modulates space use: females with dependent offspring often restrict movements to reduce infanticide risk and energetic costs, whereas single (non‐lactating) females generally maintain intermediate home range sizes—larger than those of females encumbered by cubs yet still smaller than those of males (Penteriani et al. 2025; Zarzo‐Arias et al. 2025). During the mating season, receptive females can show temporary increases in displacement or excursions that facilitate mate encounter; however, at the population level, male mate‐search behaviour remains the principal driver of the pronounced sex difference in home range area (Dahle and Swenson 2003; Bogdanović, Hertel, Plećaš, et al. 2021). At the same time, general allometric theory and comparative analyses across mammals predict a positive relationship between body mass and home range size, reflecting higher energetic requirements and lower population densities of larger individuals and species (Reiss 1988; Ofstad et al. 2016; Joly et al. 2022).

Brown bears exhibit strong sexual size dimorphism: adult males are typically 30% or more heavier than females and have larger linear body dimensions and cranial measurements (Hilderbrand et al. 1999; McDonough and Christ 2012; Burton et al. 2018). This dimorphism may contribute mechanistically to sex differences in home range through both energetic and behavioural pathways, such as larger males requiring larger areas to meet energy demands and using wider movement kernels during mating (Reiss 1988; Dahle and Swenson 2003; Katajisto and Moilanen 2006). Within‐population evidence on whether individual body mass and morphometrics (e.g., body length, chest circumference, foot size, or skull metrics) explain variation in home range size beyond the effect of sex remains limited, especially in populations at the southern edge of the species' range, such as Türkiye and the Caucasus (Burton et al. 2018; IUCN 2016). Populations at lower latitudes frequently occupy warmer, more human‐modified and fragmented landscapes, where human footprint, forest disturbance, and the distribution of anthropogenic foods can constrain or redirect movements and thus reshape the determinants of home range size. In such settings, bears may maintain smaller or differently configured ranges than expected from morphology alone, because space use is strongly modulated by human pressure, habitat configuration, and seasonal resource patchiness (e.g., across Europe and in southeastern Europe) (Hertel et al. 2025). These southern‐range contexts therefore provide a critical test of whether within‐population relationships between body size and home range area persist once anthropogenic and habitat factors dominate movement decisions (De Angelis et al. 2021; Bogdanović, Hertel, Plećaš, et al. 2021; Hertel et al. 2025). Understanding how sex and body size together shape space use is important for predicting habitat requirements, connectivity needs, and vulnerability to human disturbance for different demographic groups (Mangipane et al. 2018; Ofstad et al. 2016; Burton et al. 2018). Home range size can be quantified using different methodological frameworks, each capturing complementary aspects of animal space use. Minimum convex polygons (MCPs) remain widely used because they are simple, transparent, and comparable across studies, especially for large carnivores with long research histories (Calenge 2006). However, MCPs do not incorporate the intensity of space use and are sensitive to outliers. Kernel utilisation distributions (KUDs), by contrast, provide a probability‐based estimate of space use that identifies core and peripheral areas and is more robust to occasional long‐distance movements (Worton 1989; Katajisto and Moilanen 2006; Walter et al. 2015). Because these methods rely on different assumptions, they may yield divergent estimates when movement patterns are highly heterogeneous or when individuals vary in range shape or exploration behaviour. Including both MCP95 and KUD95 in our study allowed us to assess whether sex‐ or size‐related patterns were consistent across estimators and to evaluate the robustness of our conclusions to methodological choice.

Our primary objective was to determine whether sex or body size is the more influential intrinsic determinant of home range size within a single brown bear population. Secondarily, we evaluated whether any sex × size interaction exists. We treated estimator choice and sampling effort as robustness checks rather than focal hypotheses. In this study, we tested three linked hypotheses about sex and body size as drivers of space use. First, males would have larger seasonal home ranges (MCP and kernel 95%) than females, consistent with mating‐related ranging and previous work in Europe (Dahle and Swenson 2003; Bogdanović, Hertel, Plećaš, et al. 2021). Second, after accounting for sex, home range size would increase with body mass and other indicators of overall size (body length, chest circumference), as predicted by allometric theory and comparative analyses (Reiss 1988; Ofstad et al. 2016; Joly et al. 2022). Third, the scaling of home range with mass could differ between sexes (sex × mass interaction), reflecting sex‐specific energetic or behavioural constraints (Dahle and Swenson 2003; Mangipane et al. 2018). We also contrasted general size metrics with more specialised morphological traits to evaluate which measurements best explain within‐population variation in home range area (Hilderbrand et al. 1999; McDonough and Christ 2012).

## 2 Materials and Methods

The study used data from 73 free‐ranging brown bears ( *Ursus arctos* ) equipped with GPS collars that recorded locations at regular intervals of 1 h, studied from 2012 to 2025 by the KuzeyDoğa NGO in the northeastern part of Türkiye, in the Sarıkamış forests‐Allahuekber Mountains National Park (Akküçük and Şekercioğlu 2016; Cozzi et al. 2016). The study area is in the Kars province of northeastern Türkiye (40.191°–40.455° N, 42.395°–42.758° E), a part of the country with insufficient protected areas and where wildlife is under great pressure (Şekercioğlu et al. 2011). These habitats are home to Brown bears ( *Ursus arctos* ), Grey wolves ( *Canis lupus* ), and Caucasian lynxes (*Lynx lynx dinniki*) (Chynoweth et al. 2015; Capitani et al. 2016) with a relatively high level of large‐carnivore‐related human‐wildlife conflict (Kusak and Şekercioğlu 2021). Villagers living in areas around forests and organisations such as forest management authorities routinely use these fragmented Scotch pine forest patches, with a total area of 338.5 km^2^, for logging (Şekercioğlu et al. 2011). The ambient temperature usually does not fall below 20°C during the warm season (May–October), but it can drop below −10°C during the cold months (November–April). Mixed beech‐fir forest, mostly *Fagus sylvatica* , *Abies alba* , *Picea abies* , *Quercus* sp., *Castanea sativa* , and *Pinus* sp., constitute the forest community type in the study area (Atalay 1983). At capture, each individual was sexed, and a standardised suite of morphometric measurements was collected, including body mass, body length (excluding tail), tail length, head length, shoulder height, head and chest circumferences, distance from eye to nose, distance between ears, ear length, multiple cranial canine distances, and detailed measurements of front and hind feet (widths, pad lengths with and without claws, and central claw lengths), as well as the length of the os penis in males and vulva in females. These measurements follow common protocols in brown‐bear field studies and provide proxies for overall body size, condition, and functional morphology (Hilderbrand et al. 1999; McDonough and Christ 2012). Captured animals were then equipped with Vectronic Aerospace GPS‐GSM/Iridium collars (Vectronic Aerospace GmbH, Berlin, Germany) with two‐axis activity sensors, which continuously recorded the acceleration and stored the values within a range of 0–255 in five‐minute intervals. For each tracked individual, morphometric data were linked to movement data via a unique AnimalID code. All individuals were captured once and measured at first capture. Captures occurred between 2012 and 2025, primarily during April–June, providing broadly comparable seasonal timing across years. Because body size can vary seasonally (e.g., pre‐ vs. post‐hyperphagia), we restricted captures to this window and treated first‐capture morphometrics as each bear's intrinsic size for analysis. Body mass was log‐transformed because home range area is expected to scale allometrically with size, implying a multiplicative rather than linear relationship (Jetz et al. 2004). The log transformation also reduced skewness and improved model residual structure (linearity and homoscedasticity), making log (body mass) more suitable for inference than the untransformed variable (Curran‐Everett 2018).

### 2.1 Ethics Statement

All capture, handling, and monitoring procedures were approved by the relevant Turkish institutional and governmental authorities (Republic of Türkiye Ministry of Agriculture and Forestry, Permit No. 72784983‐488.04‐114100 and E‐21264211‐288.04‐1602322). and conducted in accordance with established guidelines for the welfare of large carnivores in the field.

### 2.2 GPS Data Processing

Only high‐quality fixes were retained for home range analysis: locations with missing coordinates were removed, and exploratory filters based on DOP and fix type were applied to exclude clearly unreliable positions, following recommended practice for GPS‐based home range estimation in large mammals (Arthur and Schwartz 1999; Walter et al. 2015). For methodological development, the full workflow was first tested on a single bear (BTR002) and then generalised to all individuals. To classify location quality, we applied explicit filters to remove unreliable GPS fixes. Fixes for lacking coordinates or timestamps were removed. We retained only locations with three‐dimensional (3D) or high‐quality two‐dimensional (2D) fixes, and excluded all low‐quality or invalid fix types (0D/no‐fix or manufacturer‐coded error flags). Horizontal dilution of precision (HDOP) values greater than 5 were discarded, as were fixes with DOP fields missing or clearly erroneous. We also removed locations implying biologically unrealistic movement between successive fixes: specifically, steps requiring speeds exceeding 10 km/h over intervals of 30 min or more, or step lengths greater than two to three times the individual's 99th percentile step length (suggesting multipath error or drift). Fixes with altitude values clearly outside plausible elevation ranges for the study area or with coordinate jumps greater than 1 km occurring within 5 min or less were also excluded. Together, these criteria ensured that retained GPS positions met spatial‐accuracy standards appropriate for home range estimation in large carnivores.

### 2.3 Home Range Estimation

The present study uses GPS collar data and detailed morphometric measurements from a sample of collared brown bears to estimate individual home range size and test how it relates to sex, body mass, and body dimensions. Home ranges are quantified using minimum convex polygon (MCP) and kernel utilisation distribution estimators, following established protocols for brown bears and other large carnivores (Arthur and Schwartz 1999; Katajisto and Moilanen 2006; Joly et al. 2022). We then link these home range estimates to a suite of morphological variables measured at capture, including mass, body length, chest circumference, shoulder height, and paw and skull metrics, to evaluate which aspects of body size and form best predict spatial requirements within this population (Hilderbrand et al. 1999; McDonough and Christ 2012). By focusing on a single population with known sex, age class, and fine‐scale movement data, the study provides individual‐level insight into the mechanisms behind sex and size‐dependent space use in brown bears, complementing broader cross‐species and cross‐population analyses (Ofstad et al. 2016; Mangipane et al. 2018). Home range size was estimated from GPS locations using the R package *adehabitatHR*, which implements widely used methods for animal space use analysis (Calenge 2006; Katajisto and Moilanen 2006). We projected WGS84 coordinates to an equal‐area coordinate reference system (Albers equal‐area projection with standard parallels bracketing the main latitudinal extent) and created per‐individual point layers for area computation. This ensured that area calculations were not biased by map projection distortions (Calenge 2006; Gurarie et al. 2017). Because our home range analyses were based on annual or active‐season ranges rather than separate seasonal estimates, we did not include season as a predictor in the models. Future work partitioning movements into biologically relevant seasons (e.g., pre‐mating, mating, post‐mating periods) would allow a clearer evaluation of energetic versus reproductive drivers of male space use.

Two complementary estimators were used. First, a 95% minimum convex polygon (MCP95) was calculated for each bear, representing the smallest convex polygon containing 95% of the locations and allowing direct comparison with earlier brown bear studies (Arthur and Schwartz 1999; Dahle and Swenson 2003). Second, a kernel utilisation distribution (KUD95) was estimated using the reference smoothing parameter (href), and the 95% volume contour was extracted to represent the probabilistic home range (Katajisto and Moilanen 2006; Walter et al. 2015). Areas of MCP95 and KUD95 were calculated in square metres and converted to square kilometres. For the present analyses, the MCP95 area in km^2^ (MCP) was used as the primary response variable because it is simple, transparent, and comparable across individuals and with the broader carnivore literature, while KUD95 results were used to verify that patterns were consistent across estimators (Arthur and Schwartz 1999; Calenge 2006).

### 2.4 KUD95 Usability Criteria and Diagnostics

To ensure that kernel utilisation distributions reflected genuine space use rather than sampling artefacts, we applied the following a priori acceptance criteria at the individual‐season level: (i) data sufficiency: at least 150 usable GPS fixes spanning more than or equal to 45 days within the respective season; (ii) temporal regularity: median fix interval less than or equal to 6 h (after filtering), and no gaps more than 10 consecutive days; (iii) bandwidth stability: the reference bandwidth (href) had to fall within 0.5–3.0 times of the median nearest‐neighbour distance among fixes; (iv) convergence: the KUD95 area versus cumulative‐fix curve had to plateau (absolute slope less than 1% change in KUD95 per additional 50 fixes); and (v) shape diagnostics: the 95% isopleth could not be dominated by outliers (operationally, more than 10% of the kernel mass lying outside a 1 km buffer of the point cloud's convex hull, or more than five disjoint patches in the 95% isopleth). Individuals failing any criterion were excluded from KUD‐based summaries and models but retained for MCP95 to preserve sample size. Rasterization used a 250 m grid with a 10 km study‐area buffer to minimise edge effects (Worton 1989; Borger et al. 2006; Laver and Kelly 2008; Kie et al. 2010; Walter et al. 2011).

To ensure comparability among individuals, home range estimation was conducted for a consistent biological period (e.g., annual or active‐season range) per bear, and animals with insufficient high‐quality fixes or very short tracking durations were excluded from the final dataset. For each retained individual, the number of locations and tracking duration (days) were recorded and later considered as potential covariates or offsets in statistical models, following recommendations on sampling effects in home range analyses (Arthur and Schwartz 1999; Walter et al. 2015).

### 2.5 Construction of the Analytical Dataset

The home range results were merged with morphometric data into a single data frame with one row per individual bear. Variables included MCP (km^2^) and KUD95 (km^2^), sex, body mass (kg), body length (cm), chest circumference (cm), shoulder height (cm), key foot, cranial metrics, and code fields linking individuals to capture events. As many detailed measurements (e.g., left/right and upper/lower canine distances, individual claw lengths) were highly correlated proxies for overall size, they were initially inspected for distribution and collinearity using correlation matrices and scatterplots. Following previous work highlighting the dominant role of general body size and mass in space use (Reiss 1988; Ofstad et al. 2016; Mangipane et al. 2018), subsequent models focused on sex, body mass, and a small set of representative linear size measures (body length, chest circumference, shoulder height), while the finer‐scale measurements were retained for exploratory or multivariate analyses but not included simultaneously as separate predictors in the main regression models. For individuals tracked across multiple active seasons, we estimated home ranges per bear‐year and treated morphometric measurements as fixed, individual‐level covariates (from first capture). This approach avoids reusing the same size data within a year while allowing repeated home range observations per individual (Fieberg and Börger 2012).

### 2.6 Statistical Analysis

Because home range areas were right‐skewed, MCP was log‐transformed before modelling to better meet linear‐model assumptions. We emphasise that the linear model's assumptions pertain to the residuals, not the raw response. We selected a log transformation of MCP after inspecting residual diagnostics on both the original and log scales: log(MCP) substantially reduced right‐tailed residuals and heteroscedasticity, yielding approximately constant variance and symmetric residuals. In addition, the log scale provides a multiplicative interpretation appropriate for home range areas that span an order of magnitude: coefficients can be interpreted as proportional (percent) differences in MCP for a one‐unit change in a predictor (holding other variables constant) (Curran‐Everett 2018). Because the model is linear on the log scale, a one‐unit increase in a predictor corresponds to a constant proportional change in MCP (i.e., diminishing absolute differences as MCP increases). This was intentional: our interest was in relative differences among individuals and sexes rather than absolute area increments, which are scale‐dependent. Where helpful, we back‐transform predictions and report effects as ratios (e.g., male: female) with 95% confidence intervals.

First, simple comparisons of home range size between sexes were conducted using boxplots and two‐sample tests, and by fitting a basic linear model with log(MCP) as the response and Sex as the sole predictor, to test the hypothesis of larger male ranges (Dahle and Swenson 2003; Bogdanović, Hertel, Plećaš, et al. 2021). Second, multiple regression models were constructed to test for effects of body mass and body size on home range, including Sex, log(body mass), and one or more linear size measures as fixed effects, with optional inclusion of tracking duration and number of fixes as covariates. We adopted an information‐theoretic (IT) workflow designed for inference from a predefined candidate set. Candidate models represented alternative biological hypotheses about sex, body mass, linear size metrics, and (where specified) Sex × Mass interactions (Burnham and Anderson 2002; Burnham and Anderson 2004; Tredennick et al. 2021). We compared models using AICc, reported ΔAICc, Akaike weights (*w*_i_), and evidence ratios, and obtained model‐averaged parameter estimates with unconditional 95% Cis (Burnham and Anderson 2004; Stanton‐Geddes et al. 2014; Tredennick et al. 2021). This approach avoids null‐hypothesis significance testing (NHST) and *p*‐values and bases inference on the relative weight of evidence and effect sizes (Burnham and Anderson 2002; Burnham and Anderson 2004; Tredennick et al. 2021). We initially considered including Age (years) as a covariate; however, age was strongly correlated with body mass and PC1 in our dataset and substantially reduced the analysable sample size (aged individuals only). To avoid collinearity and loss of power, age was excluded from the main models. In sensitivity analyses restricted to aged individuals, including age did not alter the sign or significance of the sex effect on log(MCP).

To evaluate robustness to the choice of scale and link, we also fitted (i) Gaussian‐identity models to untransformed MCP with heteroscedasticity‐consistent (HC) standard errors, and (ii) a Gamma GLM with a log link. In both alternatives, residual patterns were acceptable for inference, and the direction, magnitude, and statistical significance of the sex effect were consistent with results from the log‐linear model (White and MacDonald 1980). Thus, our conclusions about sex differences (and the weak within‐sex effects of size metrics) do not depend on the log transformation.

To examine whether the scaling of home range size with body mass differed between sexes, interaction terms between sex and log(body mass) were added and tested in extended models. Where sample sizes and model diagnostics allowed (according to the KUD95 usability criteria above), we also fitted analogous models with KUD95 km^2^ as the response to assess robustness to estimator choice (Arthur and Schwartz 1999; Katajisto and Moilanen 2006; Calenge 2006; Walter et al. 2015). We did not use α‐level decisions or *p*‐values. Inference focused on model plausibility (ΔAICc, *w*_i_), evidence ratios, and the magnitude and precision of (model‐averaged) effects with 95% confidence intervals, interpreted in a biological context. Model assumptions were evaluated using standard diagnostic plots. For each linear model, residuals versus fitted values and scale–location plots were inspected to assess homoscedasticity and potential nonlinearity, and normal Q–Q plots of residuals were used to check the approximate normality of errors. Residuals versus leverage and Cook's distances were examined to identify high‐leverage and influential observations. To assess robustness to the choice of scale and link, we fitted (i) Gaussian‐identity models to untransformed MCP with heteroscedasticity‐consistent (HC) standard errors, and (ii) a Gamma GLM with a log link. In both alternatives, residual patterns were acceptable for inference, and the direction, magnitude, and statistical significance of the sex effect were consistent with results from the log‐linear model. Thus, our conclusions about sex differences (and the weak within‐sex effects of size metrics) did not depend on the log transformation.

Distributional comparisons between sexes: To assess whether sex differences extended beyond mean home range size, we compared the dispersion and entire distributions of log‐transformed MCP areas between sexes. We used the Fligner–Killeen test (robust to non‐normality) to assess heterogeneity of variance and reported interquartile ranges (IQRs) as descriptive measures of spread. We also applied a permutation‐based energy‐distance two‐sample test (10,000 permutations) to compare the full marginal distributions of log(MCP). Finally, we fitted quantile regressions at *τ* = 0.25, 0.50, and 0.75 to examine whether the male–female contrast varied across the distribution, complementing mean‐based linear models (Koenker and Bassett Jr. 1978). All analyses and diagnostics were conducted in R 4.4.2 (R Core Team 2025).

### 2.7 Analysing Morphometric Traits

To reduce the dimensionality of the morphometric dataset and derive composite indices of overall body size and shape, a principal component analysis (PCA) was performed on eight quantitative traits: body mass, body length, chest circumference, shoulder height, and widths of the front and hind feet (left and right). Age was determined from a vestigial premolar extracted at capture and aged by cementum annuli. All variables were first converted to numeric form, inspected for plausibility, and standardised (mean‐centred and scaled to unit variance) before analysis to place them on a comparable scale. Individuals with missing values in any of the selected traits were excluded from the PCA, resulting in a complete‐case sample. The PCA was conducted on the correlation matrix, and components were retained and interpreted based on eigenvalues, proportion of explained variance, and biological interpretability of loadings. The first principal component (PC1), which had positive and similar loadings for all traits and explained about 70% of the total variance, was interpreted as a general body‐size axis. The second principal component (PC2), explaining a further ~11% of variance and contrasting linear dimensions with paw widths, was interpreted as a shape/proportionality axis. Individual PC scores for PC1 and PC2 were extracted and merged with the home range dataset and used as alternative multivariate predictors of home range size in linear models of log(MCP) together with sex.

## 3 Results

Home range size in the 69 GPS‐collared brown bears varied widely, from 5.1 to 203.9 km^2^ (median 70.5 km^2^, mean 81.5 km^2^). The sample was male‐biased, with 46 males and 23 females. Body mass ranged from 50.5 to 260.0 kg (mean 139.8 kg), with five individuals lacking reliable mass measurements. The MCP95 home range area was right‐skewed, so all analyses used log‐transformed MCP values.

### 3.1 Body Size and Morphometrics

Correlations among morphometric variables indicated a strong common body‐size axis. Body mass was highly correlated with chest circumference (*r* = 0.93) and strongly correlated with both body length (*r* = 0.75) and shoulder height (*r* = 0.69). Body length, chest circumference, and shoulder height were also positively intercorrelated (*r* = 0.66–0.72). These patterns confirmed that the measured morphometrics (body mass, body length, chest circumference, and shoulder height) capture overall size and condition, consistent with pronounced sexual size dimorphism, and justified treating body mass, body length, and chest circumference as representative size metrics. The following table (Table 1) summarises this information.

**TABLE 1:** The correlation between various morphological characteristics of the captured bears.

Variable	Mass (kg)	Body length (cm)	Chest circumference (cm)	Shoulder height (cm)
Mass (kg)	1.00	0.79	0.93	0.71
Body length (cm)	0.79	1.00	0.76	0.69
Chest circ. (cm)	0.93	0.76	1.00	0.73
Shoulder height (cm)	0.71	0.69	0.73	1.00

**FIGURE 1:**
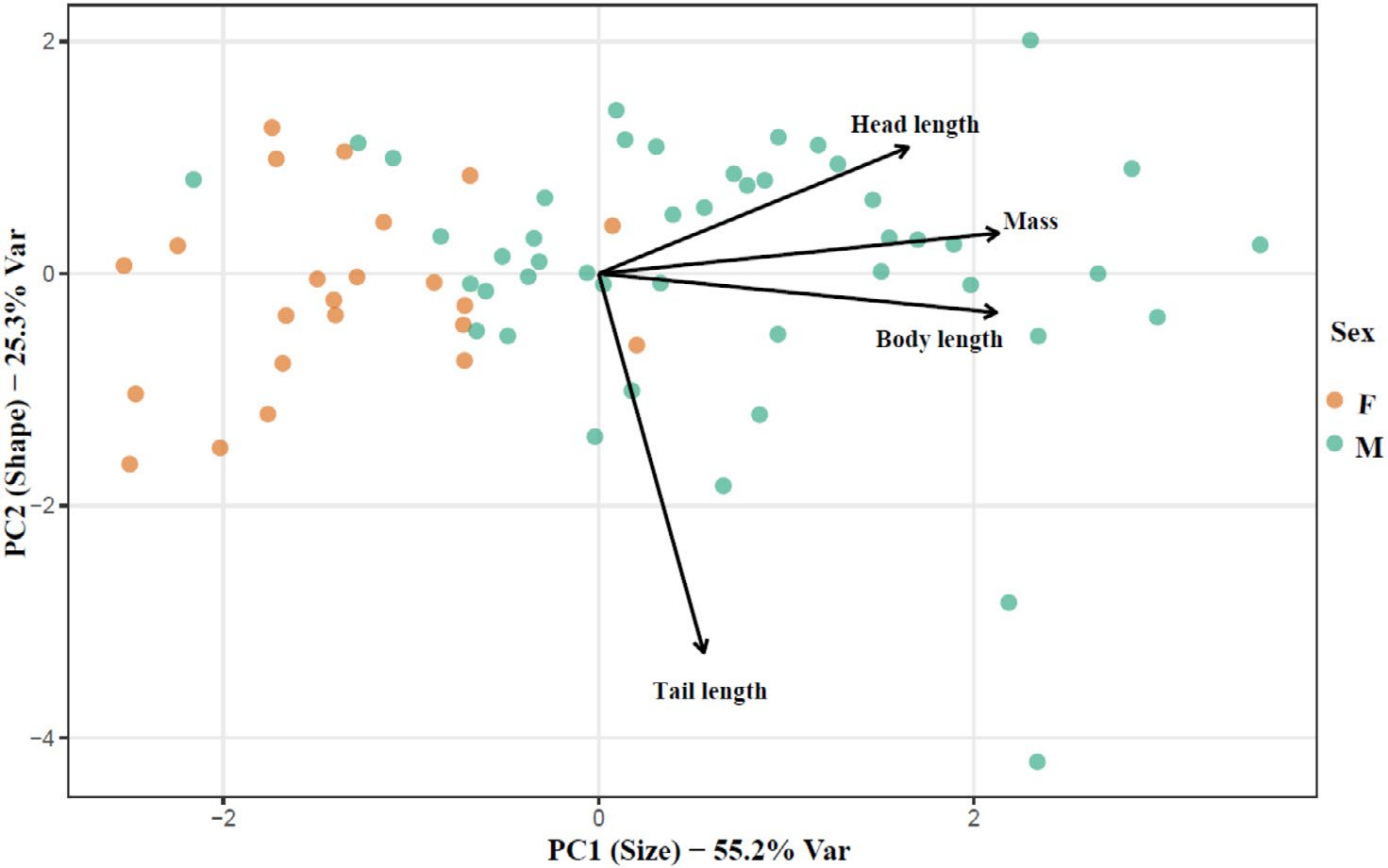
Principal Component Analysis (PCA) biplot of brown bear morphometric traits. The plot displays individual bear scores along the first two principal components, with symbols colour‐coded by sex (green = male; orange = female). Vectors represent the variable loadings for body mass, body length, head length, and tail length; vector length corresponds to the strength of the contribution to the variance, and the angle between vectors indicates the correlation between traits. PC1 represents an overall size gradient, while PC2 represents a distinct component of body shape. Only individuals with complete measurements for these four variables were included in the analysis.

To summarise morphological variation, a Principal Component Analysis (PCA) was conducted on body mass, body length, head length, and tail length (all standardised). PC1 explained 55.2% of the total variance and loaded positively on all traits—particularly mass and body length—representing an overall body‐size axis. PC2 explained 25.3% of the variance and was primarily driven by tail length, representing a distinct component of body shape (Figure 1). When PC1 and PC2 were included in a linear model with log‐transformed MCP home range size as the response, sex remained the only significant predictor (estimate for males = 0.77 ± 0.28 SE, *p* = 0.0087). In contrast, neither the size‐related PC1 (−0.05 ± 0.06 SE, *p* = 0.36) nor the shape‐related PC2 (0.16 ± 0.10 SE, *p* = 0.12) was a significant predictor (Tables 2A, 2B). These results indicate that multivariate body‐size and shape components did not explain additional variation in home range size beyond the categorical effect of sex.

**TABLE 2A:** Model selection results. This table provides the ‘evidence’ for your conclusions. It shows that you didn't just guess; you mathematically compared multiple hypotheses.

Model	Predictors	K	AICc	ΔAICc	Weight (*w*_i_)	*R* ^2^
Sex	Sex	3	165.27	0.00	0.51	0.00
Additive	Sex + log(Mass)	4	166.44	1.18	0.28	0.14
Interaction	Sex * log(Mass)	5	167.32	2.06	0.18	0.04
Mass	log(Mass)	3	173.15	7.88	0.01	0.15
Null	1	2	173.42	8.16	0.01	0.00

**TABLE 2B:** Parameter estimates for the best‐supported model (Sex model). This table provides the specific ‘effect size’ of the variable that actually matters (Sex).

Term	Estimate	Std. error	*t*	*p*
Intercept	3.68	0.16	23.00	< 0.001
Sex (Male)	0.65	0.20	3.29	0.0016

To clarify the relationship between the PCA results and the univariate morphometric analyses, the PCA was used to test whether multivariate axes of body form improved the prediction of home range size beyond sex. Although PC1 captured over half of the morphological variance, it did not improve model support once sex was included. For comparability with previous brown‐bear studies and allometric predictions (Swenson et al. 2007; Evans et al. 2023; Harestad and Bunnell 1979; Lindstedt et al. 1986; Jetz et al. 2004; Ofstad et al. 2016), which are conventionally expressed via body mass, we also evaluated representative single traits directly. Importantly, the conclusions remained consistent across approaches: neither the PCA‐derived composite size metrics nor individual morphometric variables improved model support relative to the sex‐only model.

### 3.2 Sex Differences in Home Range Size

**FIGURE 2:**
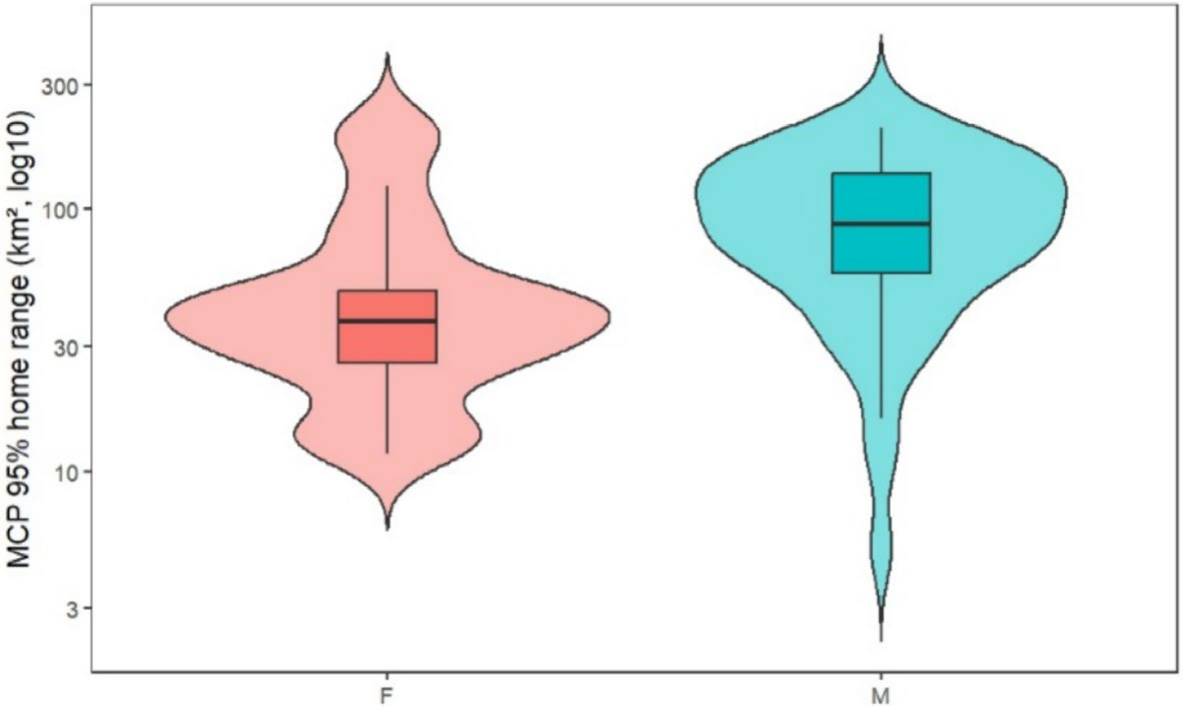
Distribution of MCP95 home range size (km^2^, log_10_ scale) for male and female brown bears, shown as violin plots with superimposed boxplots. The violin plot illustrates the full distribution of values for each sex, while boxplots display medians and interquartile ranges. Internal grid lines have been removed for visual clarity.

Home range sizes were highly variable, with arithmetic means of 54.2 km^2^ for females and 95.1 km^2^ for males. However, models based on log‐transformed data—which better account for the skewed distribution of space‐use data—indicated that the typical female home range was approximately 40 km^2^, while the typical male home range was 76 km^2^ (a significant increase of 0.65 log‐units; *t* = 3.29, *p* = 0.0016). Back transformation indicates that males used, on average, 1.92 times larger MCP95 home ranges than females. Sex alone explained 14% of the variance in log home range size among individuals (*R* ^2^ = 0.14, adj. *R* ^2^ = 0.13; *F*_1, 67_ = 10.84, *p* = 0.0016), supporting the hypothesis that males maintain substantially larger spatial requirements than females in this population (Figure 2).

### 3.3 Effects of Body Mass and Body Size

**FIGURE 3:**
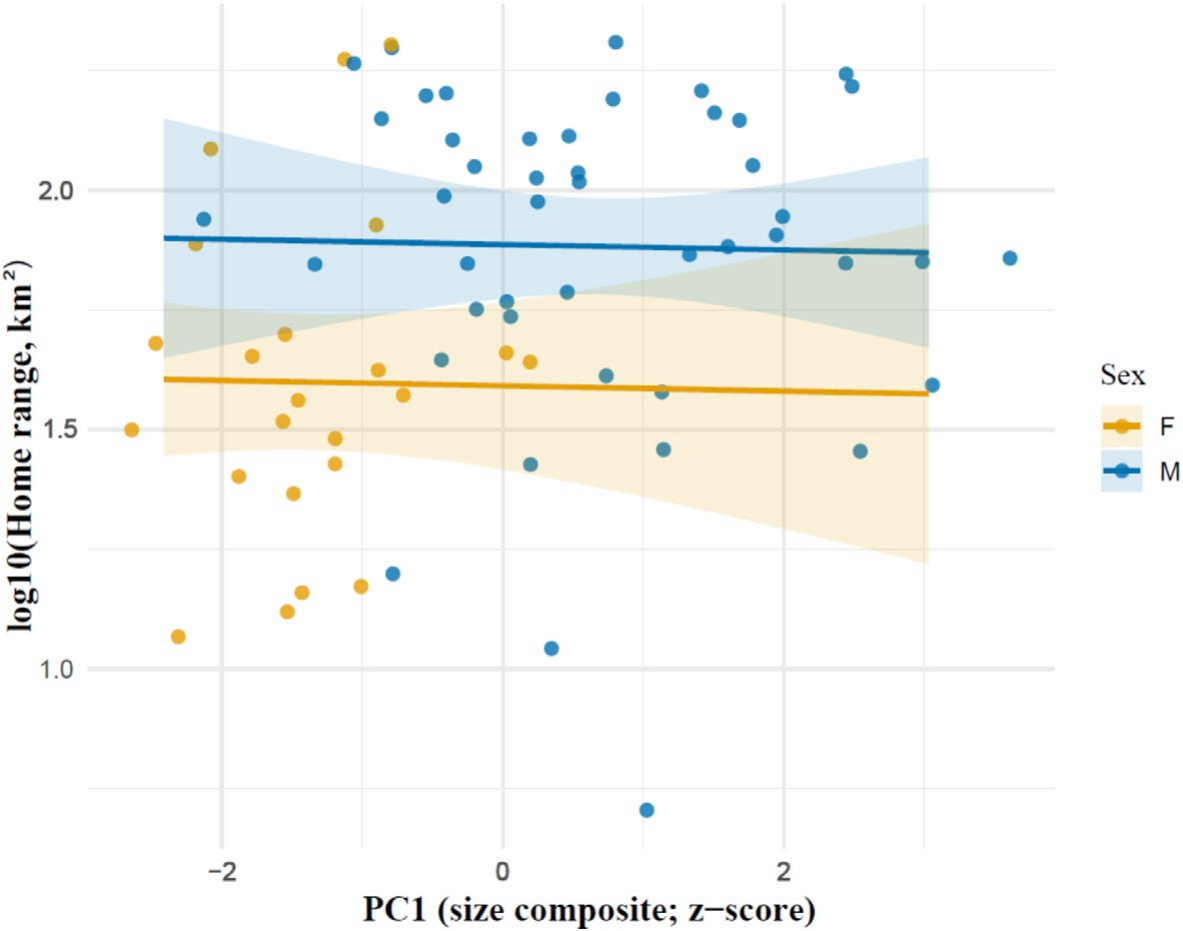
Relationship between home range size and a morphometric composite (PC1) in GPS‐collared brown bears. PC1 was derived using a complete‐variable PCA (Option A), which retained only morphometric variables with no missing values and therefore included individuals missing a single measurement (e.g., mass). Points show individual bears coloured by sex. Lines represent model‐based predictions from a linear model of log_10_(home range area) on PC1 and sex (no interaction), with shaded ribbons indicating 95% confidence intervals. PC1 showed no detectable association with home range size (*β* = −0.006 ± 0.037 SE, *p* = 0.881), whereas males exhibited substantially larger home ranges than females (*β* = 0.295 ± 0.116 SE, *p* = 0.013). Sample size: *N*≈69.

Including body mass and body length did not materially alter the sex effect and did not reveal significant effects of body size on home range area (Figure 3). In this model, males still had significantly larger home ranges (sex coefficient = 0.85 ± 0.31 SE, *t* = 2.79, *p* = 0.0070), but log body mass (estimate −0.33 ± 0.49 SE, *t* = −0.67, *p* = 0.503) and standardised body length (−0.03 ± 0.16 SE, *t* = −0.17, *p* = 0.869) were not significant predictors. The model explained about 15% of the variance in log(MCP) (*R* ^2^ = 0.15, adj. *R* ^2^ = 0.11; *F*_3, 60_ = 3.49, *p* = 0.021), using 63 individuals (the remainder were excluded due to missing mass or length values).

Replacing body length with chest circumference (model M3) produced very similar results. Males again had significantly larger home ranges (sex coefficient = 0.80 ± 0.31 SE, *t* = 2.59, *p* = 0.012), whereas log body mass (0.25 ± 0.67 SE, *t* = 0.37, *p* = 0.717) and standardised chest circumference (−0.28 ± 0.25 SE, *t* = −1.10, *p* = 0.276) were not significant. This model explained 16% of the variance in log(MCP) (*R* ^2^ = 0.16, adj. *R* ^2^ = 0.11; *F*_3, 58_ = 3.60, *p* = 0.018; *n* = 62). Thus, once sex was accounted for, within‐sex variation in body mass and simple linear dimensions did not meaningfully explain additional variation in home range size.

The Sex × log(body mass) model was not competitive (ΔAICc = 4.60; *w*_i_ = 0.10) compared to the best model (sex‐only; ΔAICc = 0.00; *w*_i_ = 0.98) or to additive models that included mass and linear size metrics (ΔAICc = 2.10–5.20; *w*_i_ = 0.08–0.21). Accordingly, we find no support for sex‐specific mass scaling of home range area and proceed with additive models. Thus, there was no evidence that the scaling of home range size with body mass differed between males and females, and subsequent inference relied on the simpler additive model, in which sex remained the only significant predictor of log(MCP).

**FIGURE 4:**
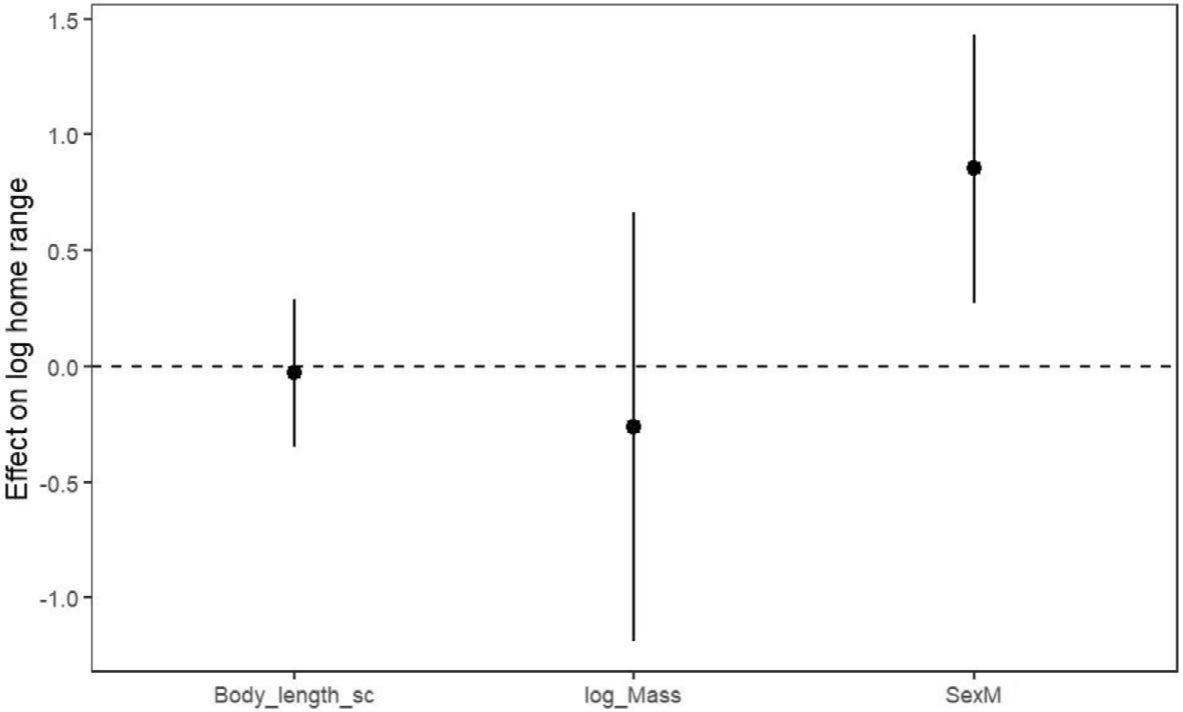
Estimated effects of sex and body‐size predictors on log‐transformed MCP95 home range sizes from linear model M2. Points represent regression coefficients with 95% confidence intervals; the dashed horizontal line indicates no effect. The effect of being male is clearly positive (confidence interval entirely above zero), whereas the effects of body length and log body mass are weak and their confidence intervals overlap zero.

When sampling effort was included as a covariate, sex remained the strongest predictor of home range size (Figure 4). In a model with sex, log body mass, standardised body length, log number of fixes, and tracking duration, males still had significantly larger home ranges than females (estimate = 0.95 ± 0.28 SE, *t* = 3.44, *p* = 0.0011). The log number of GPS fixes showed a positive association with log home range size (0.16 ± 0.06 SE, *t* = 2.54, *p* = 0.014), indicating that individuals with more locations tended to have slightly larger estimated ranges, whereas tracking duration, body mass, and body length remained non‐significant. This extended model explained about 30% of the variation in log(MCP) (*R* ^2^ = 0.36, adj. *R* ^2^ = 0.30; *F*_5, 55_ = 6.12, *p* < 0.001), confirming that the sex effect is not an artefact of differential sampling effort. Diagnostic plots and Cook's distances identified five potentially influential individuals, but excluding these did not change the main conclusions. The inclusion of sampling effort covariates improved overall model fit (*R* ^2^ = 0.36). Both the log‐transformed number of GPS fixes (0.16 ± 0.06 SE, *p* = 0.014) and tracking duration showed positive associations with estimated home range size. However, even after accounting for these sampling effects, the difference between males and females remained large and statistically robust (*p* < 0.001).

We replaced mass and individual linear measurements with a morphometric composite (PC1) derived from a complete‐variable PCA (Option A), which allowed inclusion of individuals missing single measurements. In a linear model of log_10_ home range size, PC1 showed no association with home range (*β* = −0.006 ± 0.037 SE, *p* = 0.881), corresponding to an estimated multiplicative change of 0.99× per +1 SD (95% CI: 0.83–1.17). In contrast, sex had a strong positive effect, with males exhibiting approximately 1.97× larger home ranges than females (*β* = 0.295 ± 0.116 SE, *p* = 0.013; 95% CI for the multiplicative factor: 1.16–3.36). At mean body size (PC1 = 0), the predicted home range area was 39.0 km^2^ for females (95% CI: 26.1–58.4) and 77.0 km^2^ for males (95% CI: 59.5–99.8). Model fit was modest (*R* ^2^ = 0.14), and diagnostic tests indicated no heteroskedasticity (Breusch–Pagan *p* = 0.98). These results confirm that body size does not predict home range size in this dataset, while sex differences remain pronounced. The PCA approach also retained 69 individuals who would have been excluded in mass‐based or length‐based models, thereby removing the distributional distortion visible in the previous Figure 3.

### 3.4 Distributional Differences by Sex

**FIGURE 5:**
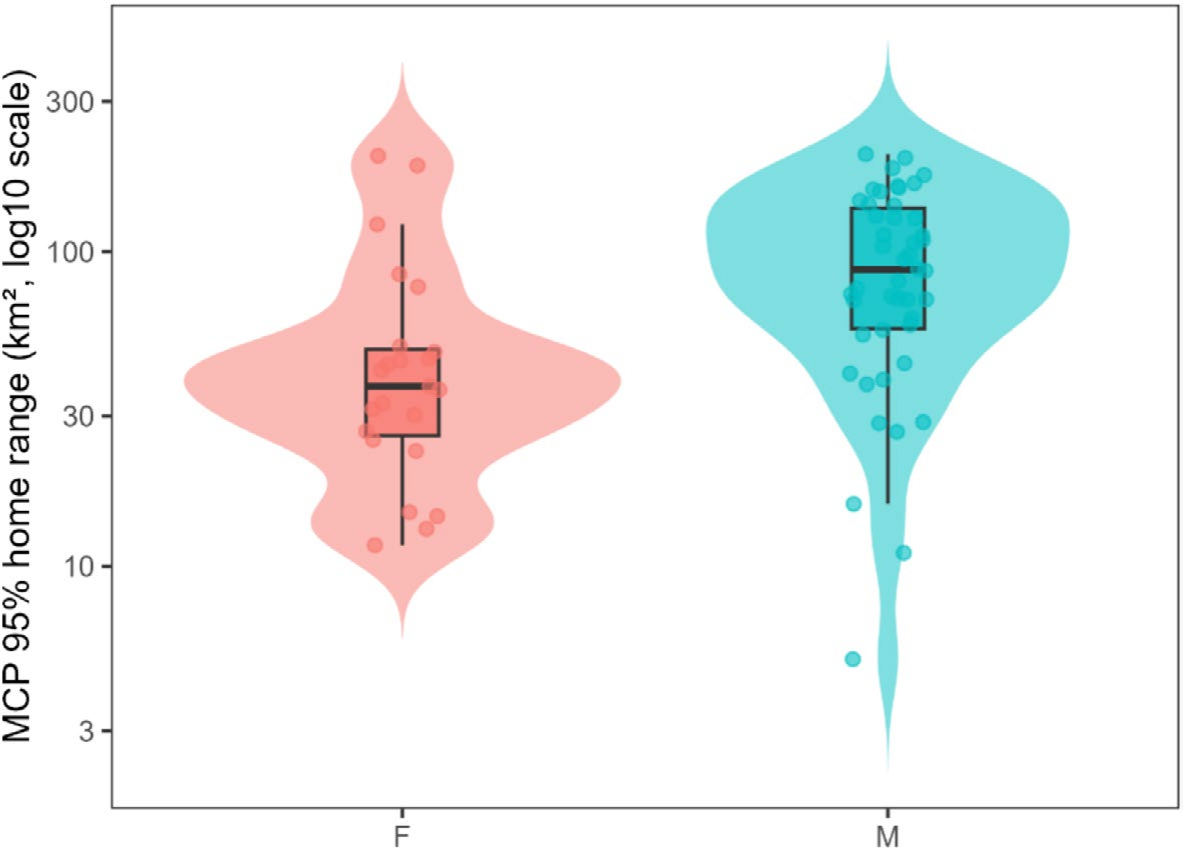
Distribution of MCP95 home range size (km^2^, log10 scale) for male and female brown bears. Violin plots display the full distribution of values for each sex, with overlaid boxplots indicating medians and interquartile ranges (IQRs). Females had an IQR of 22.9 km^2^, while males had an IQR of 80.6 km^2^, but dispersion did not differ statistically on the log scale (Fligner–Killeen test: *χ* ^2^ = 0.007, df = 1, *p* = 0.934). Although variance was similar, males showed a marked right‐shift in overall range size, consistent with quantile‐regression results indicating larger male home ranges across lower, median, and upper quantiles.

Beyond mean differences, dispersion on the log scale did not differ between sexes (Fligner–Killeen: *χ* ^2^ = 0.007, df = 1, *p* = 0.934; IQR_log: females = 0.632, males = 0.882). By contrast, a distribution‐free, permutation energy‐distance test indicated that the entire distributions of log‐transformed MCP differed between sexes (*p* = 0.0002, 10,000 permutations). Quantile regressions showed consistently positive male effects across the distribution, with larger male–female contrasts towards the upper tail (*τ* = 0.25: *β* = 0.804 [0.178, 1.431], ratio = 2.235; *τ* = 0.50: *β* = 0.847 [0.498, 1.196], ratio = 2.333; *τ* = 0.75: *β* = 1.030 [0.219, 1.841], ratio = 2.801). Together, these results indicate that while variance was comparable on the modelling (log) scale, male ranges were stochastically larger across most quantiles, with the sex gap widening towards the upper tail (Figure 5).

To evaluate the relative support for different hypotheses, we used an information‐theoretic approach based on Akaike's Information Criterion corrected for small sample sizes (AICc; Table 2A). Model selection indicated that a model including only sex as a predictor was the most parsimonious, receiving 51.3% of the total model weight. Adding body mass (additive model) or an interaction term (Sex × log(Mass)) did not improve model support (ΔAICc > 1). Based on the top‐supported model, males had significantly larger home ranges than females (*t* = 3.29, *p* = 0.0016, Estimate = 0.65 ± 0.20 SE on the log scale). These results suggest that body mass does not provide additional explanatory power for home range size variation once the categorical effect of sex is accounted for, reinforcing the primacy of sex‐related behavioural or energetic differences in this population. Model assumptions were verified through visual inspection of residual‐fitted, scale‐location, and Q–Q plots (Figure S1). The analysis confirmed no strong deviations from linearity or homoscedasticity, and errors were approximately normally distributed. Diagnostic plots for leverage and Cook's distance indicated a small number of potentially influential individuals; however, sensitivity analyses performed by excluding these outliers indicated that they did not alter the direction or statistical significance of the reported sex effect.

## 4 Discussion

The main contribution of this study is to resolve, within a single southern‐range population, that sex overwhelmingly outperforms body size as an intrinsic predictor of home range area once sampling effort is accounted for, with results robust across estimators and model scales. The analyses indicate that sex strongly structures space use in this brown bear population, whereas individual variation in body size and morphometrics adds little explanatory power once sex and sampling effort are accounted for (Dahle and Swenson 2003; Bogdanović, Hertel, Plećaš, et al. 2021; Mangipane et al. 2018). Males consistently maintained home ranges that were nearly twice as large as those of females, even after controlling for body mass, linear body dimensions, and GPS effort covariates. This pattern matches expectations from reproductive strategy theory and previous work on brown bears and other large carnivores, where males expand their movements to search for mates and overlap multiple female ranges, while females—particularly those with cubs—restrict movements to safer, resource‐rich areas (Dahle and Swenson 2003; Bogdanović, Hertel, Plećaš, et al. 2021). The magnitude of the sex effect in this study (approximately 1.8–2.0‐fold difference) falls within the range reported for other European and Arctic brown bear populations, suggesting that the population‐level drivers of sexual differences in space use are broadly consistent across contrasting environments (Dahle and Swenson 2003; Burton et al. 2018; Joly et al. 2022). Additionally, because our models focused on intrinsic traits, unmeasured environmental and landscape variables, such as habitat configuration, food distribution, and human disturbance, may contribute to the unexplained variation in home range size. These factors could partially obscure subtle morphology–space–use relationships, and incorporating spatial covariates in future analyses would provide a clearer understanding of these interactions.

In contrast to the strong sex effect, neither body mass nor simple linear measures of body size (body length, chest circumference, shoulder height) explained additional variation in home range size within each sex (Reiss 1988; Ofstad et al. 2016). Contrary to naïve allometric expectations, which predicted that larger individuals should require proportionally larger areas to meet their energetic demands, our results showed no detectable within‐sex size effect on home range area. Increasing evidence across mammals demonstrates that, within populations, behavioural and ecological processes typically overshadow fine‐scale morphological variation (Reiss 1988; Ofstad et al. 2016). In brown bears specifically, several well‐documented behavioural mechanisms decouple body size from space use. These include (i) sex‐specific reproductive strategies, where males roam widely to locate mates while females—especially those with cubs—constrain movements to reduce infanticide risk (Dahle and Swenson 2003; Steyaert et al. 2013), (ii) individual differences in foraging strategies and risk tolerance, which can generate large variation in movement even among similarly sized individuals (Mangipane et al. 2018), and (iii) strong responses to human activity, where bears adjust diel activity and movement distances to avoid people, sometimes reducing movement independently of body size (Ordiz et al. 2013; Ordiz et al. 2017). Together, these behavioural mechanisms provide a more powerful explanation for within‐population variation in space use than fine‐scale morphological differencesThe very high correlations among mass, chest circumference, body length, and shoulder height demonstrate that these traits largely capture a single size dimension and confirm strong sexual size dimorphism in the study animals, yet this dimorphism is already encoded in the categorical sex term of the models (Hilderbrand et al. 1999). Once sex is included, residual variation in body size is relatively small, and any effect of within‐sex size differences on home range is evidently weak compared with behavioural strategies, life‐history stage, or local resource heterogeneity (Mangipane et al. 2018; Burton et al. 2018).

The PCA further supports this interpretation. PC1, which explained over 70% of the variance and represented a multivariate body‐size axis with positive loadings for all morphometric traits, did not significantly predict home range size when entered alongside sex, and PC2, which captured differences in proportionality between linear dimensions and paw widths, also showed at most a weak, non‐significant association with space use (Jolliffe 2002; Budaev 2010; Reiss 1988). These results indicate that even when morphometrics are summarised into orthogonal size and shape components, they do not account for additional variation in home range area beyond the effect of sex. Across all analyses, continuous morphometric variation, whether expressed as raw measurements or as PC1, did not explain additional variation in space use once sex was included. This further supports the conclusion that sex‐linked behavioural strategies, rather than fine‐scale size variation, dominate spatial ecology in this population (Hilderbrand et al. 1999). We also evaluated the relationship between size and home range area within each sex using untransformed MCP values. These checks yielded the same conclusion as the main models: within‐sex size variation does not predict home range area. This robustness analysis confirms that the absence of a size effect is not an artefact of the log transformation or model structure.

These individual‐level processes scale up to shape broader geographic patterns in brown bear spatial ecology. Across populations, home range size and overlap shift with habitat productivity, seasonality, and density, reflecting how behavioural decisions interact with landscape structure (McLoughlin 2000). For example, access to energy‐rich foods such as salmon influences population‐level body size and productivity but does not necessarily translate into larger home ranges within a single population (Hilderbrand et al. 1999; Joly et al. 2022). Thus, our results support the growing consensus that spatial behaviour in brown bears, and many other large mammals, is governed primarily by life‐history strategies, risk, and resource heterogeneity rather than fine‐scale variation in morphology.

Analyses of sampling effort and influential points strengthen confidence in these conclusions. Males and females did not differ significantly in the number of GPS fixes, and tracking durations were only marginally longer for females. Nevertheless, sex remained the dominant predictor of home range size after explicit inclusion of log(number of fixes) and tracking duration (Arthur and Schwartz 1999; Walter et al. 2015). Our results confirm that the observed sex differences are not artefacts of sampling bias. While estimated home range size increased with tracking duration and fix frequency, a common technical relationship in telemetry studies (Arthur and Schwartz 1999; Walter et al. 2015), the sex effect remained the dominant driver. This suggests that the nearly twofold difference in space use between male and female bears in Türkiye is a biological reality reflecting divergent reproductive strategies (Dahle and Swenson 2003; Bogdanović, Hertel, Plećaš, et al. 2021) rather than a consequence of differential GPS collar performance or monitoring effort (Calenge 2006). Similarly, removing a small number of statistically influential individuals changed neither the direction nor the significance of the sex effect, indicating that the observed pattern is not driven by a few outliers. Model diagnostics suggested that linear models with log‐transformed home range area provided an adequate description of the data, with no major violations of normality or homoscedasticity (Zuur et al. 2010; Quinn and Keough 2002).

The relatively modest proportion of variance explained by the final models (adjusted *R* ^2^ around 0.11–0.33 depending on whether effort is included) is typical for movement and behavioural traits and indicates additional unmeasured drivers of space use (Ofstad et al. 2016; Mangipane et al. 2018). Habitat composition and configuration, anthropogenic features, individual behavioural syndromes (e.g., boldness, risk tolerance), and reproductive status likely contribute to the residual heterogeneity in home range size (Mangipane et al. 2018; Bogdanović, Hertel, Plećaš, et al. 2021; Burton et al. 2018). Previous studies have indicated that access to productive habitats, use of anthropogenic food sources, and landscape heterogeneity can markedly influence brown bear movements and home ranges, sometimes mediating or amplifying sex differences (Mangipane et al. 2018; Joly et al. 2022). Integrating the present morphometric and space‐use dataset with spatially explicit habitat and human activity layers would be a natural next step to disentangle how environmental context and individual traits jointly shape spatial ecology.

From a conservation and management perspective, the finding that sex, rather than fine‐scale body size, primarily determines home range size has practical implications. Planning of protected areas, movement corridors, and mitigation measures in human‐dominated landscapes should explicitly account for the larger spatial requirements of males while recognising that females, despite having smaller home ranges, may be more sensitive to local habitat quality and disturbance, especially during reproductive periods (IUCN 2016; Burton et al. 2018). The absence of a strong within‐sex size effect suggests that management can treat adult males and females as relatively homogeneous groups in terms of spatial requirements, focusing on sex‐specific rather than size‐specific strategies. At the same time, the large individual variation in home range area highlights the need for flexible, landscape‐scale planning that accommodates wide‐ranging males and more sedentary females within a connected mosaic of secure habitats (Dahle and Swenson 2003; Mangipane et al. 2018).

### 4.1 Applicability and Limitations

Brown bears inhabit environments that differ greatly in density, human impact, and resource seasonality. Our finding that sex has a greater influence than within‐sex size is likely general, as polygynous mating, infanticide risk, and human avoidance strongly influence movement. However, populations with significantly different densities or resource pulses (e.g., coastal salmon) may exhibit different absolute ranges and sex differences. Our single‐population design enhances internal validity, but limits generalisation; multi‐population meta‐analyses with standardised estimators and controlled effort are a priority. The results may have been even more distinct and convincing if GPS tracking datasets were subdivided and analysed by seasons well‐defined seasonal phases in the annual life cycle of bears, distinguishing denning, mating, and foraging seasons, which are found in all brown bear populations living in temperate and northern regions (Dahle and Swenson 2003).

### 4.2 Implications for Conservation and Management

The finding that sex, not fine‐scale body‐size variation, primarily structures home range area has important management consequences. Current management frameworks often implicitly assume size‐based space‐use differences grounded in metabolic theory; however, our results show that sex‐specific behaviour, reproductive strategy, and risk avoidance are far more relevant drivers. In Türkiye, where brown bears are legally protected, and conflicts with humans frequently occur near settlements, agricultural areas, and protected‐area edges, management strategies should therefore prioritise:

- maintaining sufficient landscape connectivity to support wide‐ranging males,
- safeguarding high‐quality, low‐disturbance habitats for females, especially during denning and cub‐rearing,
- reducing human–bear conflict zones (e.g., around apiaries, villages, and farmland) where bears concentrate movements, and,
- explicitly accounting for sex‐specific behaviours rather than assuming larger individuals require larger areas.

Because within‐sex variation in size played little role in shaping space use, management can treat adult males and females as relatively homogeneous groups regarding spatial requirements, focusing instead on behavioural ecology and human footprint as primary determinants of conflict risk and habitat needs.

## Author Contributions

**Morteza Naderi:** conceptualization (equal), data curation (equal), formal analysis (equal), funding acquisition (equal), investigation (equal), methodology (equal), software (equal), validation (equal), visualization (equal), writing – original draft (equal), writing – review and editing (equal). **Emrah Çoban:** data curation (equal), writing – original draft (equal), writing – review and editing (equal). **Uygar Can Çelik:** data curation (equal), writing – original draft (equal), writing – review and editing (equal). **Josip Kusak:** data curation (equal), formal analysis (equal), supervision (equal), writing – original draft (equal), writing – review and editing (equal). **Çağan H. Şekercioğlu:** data curation (equal), project administration (equal), resources (equal), supervision (equal), writing – original draft (equal), writing – review and editing (equal).

## Funding

Funding for this project was provided primarily by Fondation Segré and the Sigrid Rausing Trust. We also acknowledge the generous contributions of the following donors and organisations: Arkadaşlar, Bilge Bahar, Seha İşmen, Ömer Külahcıoğlu, Burak Över, Batubay Özkan, Emin Özgür, Suna Reyent, Faruk Yalçın Zoo, National Geographic Society, STGM, TANAP, TÜBİTAK (under the 219Z066 fund), Barbara Watkins, BTC Co., the Whitley Fund, Bernd Thies Foundation, UK Wolf Conservation Trust, the EU LIFE Program, and the Croatian Fund for Nature and Environment.

## Conflicts of Interest

The authors declare no conflicts of interest.

## Supporting information


**Figure S1:** Visual inspection of residual–fitted and scale–location plots indicated no strong deviations from linearity or homoscedasticity, and residual Q–Q plots showed that model errors were approximately normally distributed. Residuals versus leverage and Cook's distance plots identified a small number of potentially influential individuals, but excluding these bears did not change the direction or significance of the sex effect on home‐range size.

## Data Availability

All data generated or analysed during this study are made available at Zenodo (DOI: https://doi.org/10.5281/zenodo.19139531).
