# Supplementary material for: Why Size Does Not Matter: Sex Driven Home Range Differences in Brown Bears
Source: Ecol Evol. 2026 Apr 20;16(4):e73531. doi: 10.1002/ece3.73531 (PMC13095866; doi:10.1002/ece3.73531)
Supplement: Supplementary file 1 — Figure S1: Visual inspection of residual–fitted and scale–location plots indicated no strong deviations from linearity or homoscedasticity, and residual Q–Q plots showed that model errors were approximately normally distributed. Residuals versus leverage and Cook's distance plots identified a small number of potentially influential individuals, but excluding these bears did not change the direction or significance of the sex effect on home‐range size. [file ECE3-16-e73531-s001.docx]

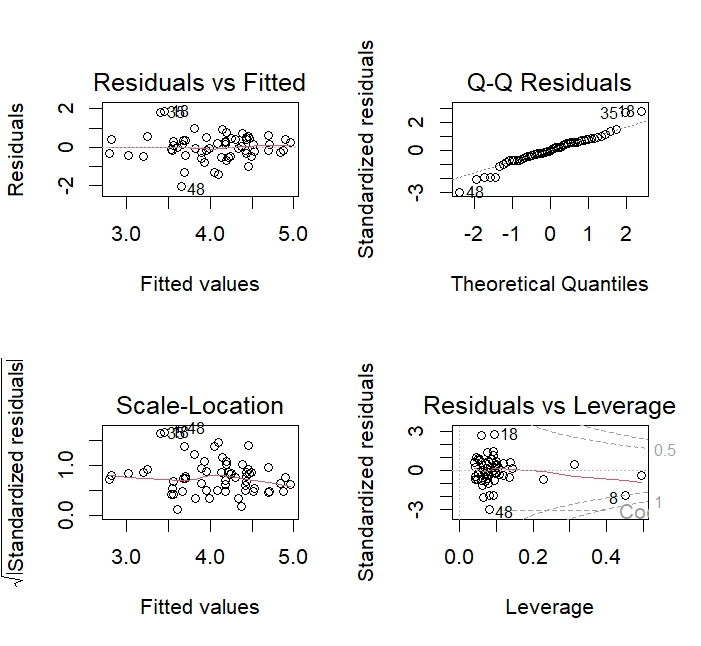


Figure S1. Visual inspection of residual–fitted and scale–location plots indicated no strong deviations from linearity or homoscedasticity, and residual Q–Q plots showed that model errors were approximately normally distributed. Residuals versus leverage and Cook’s distance plots identified a small number of potentially influential individuals, but excluding these bears did not change the direction or significance of the sex effect on home‑range size.
